# Operating Room Emergency Manuals Improve Patient Safety: A Systemic Review

**DOI:** 10.7759/cureus.4888

**Published:** 2019-06-12

**Authors:** Wayne R Simmons, Jeff Huang

**Affiliations:** 1 Anesthesiology, Hospital Corporation of America West Florida Graduate Medical Education Consortium / Oak Hill Hospital, Brooksville, USA; 2 Anesthesiology, University of Central Florida College of Medicine, Orlando, USA

**Keywords:** emergency manuals, cognitive aid, perioperative, patient safety, simulation-based training, crisis prevention, anesthesiology, perioperative medicine, operating room, crisis resource management

## Abstract

The aim of this review is to highlight the latest movements surrounding Emergency Manual (EM) implementation nationally and abroad within perioperative medicine with a focus on studies linking EM to patient safety. This is a comprehensive literature review which includes a brief introduction to the definition and history of EM as well as an overview of a successful implementation strategy, international influence and correlations to patient safety. The recent changes in healthcare and healthcare reimbursement have directed the focus throughout healthcare to quality improvement and patient safety. The potential of EMs' application to improve patient outcomes has influential implications both on patient outcomes as well as reimbursements. This study includes relevant citations with the large majority published in the last five years. EM implementation in healthcare has grown within the US and internationally over the last decade. Prominent organizations have created EMs containing principles of evidence-based medicine and widely accepted protocols that have been endorsed by major entities in the medical field. Successful implementation strategies primarily focus on different forms of simulation training and have been found to increase adherence to protocols through EM use. An increasing amount of educational institutions and healthcare facilities worldwide are perpetuating such implementation and a growing number of successful cases are being published.

## Introduction and background

What are emergency manuals?

For the purposes of this article we will not delve into any in-depth definition for the term “Emergency Manual” (EM). Other articles have been highly efficient in doing so and seem to have mostly come to a consensus, and thus, in this article we will follow the established pattern of reference to any cognitive aid or crisis manual as simply "Emergency Manual": A tool made to command all resources at hand in order to provide an anesthesia delivery plan in concert with members of the anesthesia care team and operating room personnel in the aid of dynamic decision-making [1-3]. EM are tools made to reliably optimize memory retrieval for rarely used information where the most frequent or hazardous omissions in patient care are found, as opposed to replacing the need for knowledge and continuous study [4-9]. In this article we will investigate and accentuate the relationship between EMs and the reason that they exist within healthcare, namely, to enhance patient safety.

History of emergency manuals

As the definition of EM has been established in previous articles, so has a history of their relationship to other high-stress professions and industries. The greatest and most cited example is the evolution of Crew Resource Management that has been developed in aviation over the last several decades [10]. Through the aviation industry we can see that EM has been built and developed to solve similar problems to those seen in perioperative healthcare today [11], namely human errors such as memory lapses in stressful situations, fatigue, and poor team communication. In fact, such a strong culture was built over time around EM usage in aviation that the expectation is never to rely solely on memory or vigilance for flight preparation [4,12]. EM in both aviation and healthcare have been found to strengthen team communication and provide key steps in time-sensitive moments [1].

EM development in healthcare is following a similar path that was taken in aviation starting with recognition of an area where improvement is needed. Over the last number of years there have been multiple organizations that are taking hold to a standard of increasing patient safety through EM utilization. Many different types of EM have been created and endorsed by prominent organizations including and not limited to the following: The World Health Organization, American Society of Regional Anesthesia and Pain Medicine in favor of a Local Anesthetic Systemic Toxicity checklist, Anesthesia Patient Safety Foundation in sponsoring a workshop on EM implementation, American Heart Association with various level of cardiac life support, Malignant Hyperthermia Association of the United States with a checklist specific to treatment of malignant hyperthermia, Society of Obstetric Anesthesia and Perinatology with a consensus statement on the management of cardiac arrest in pregnancy with a specific checklist development, Society of Pediatric Anesthesia in development of crisis checklists in Pediatric Care [13]. In fact, there have been entire professional organizations built for the creation, education, implementation, and research of EM. The Emergency Manual Implementation Collaborative is one of these organizations wherein the most expansive up-to-date and widely accepted perioperative EMs are provided to healthcare professionals and endorsed by the Council on Surgical and Perioperative Safety. Among the provided EM on the EMIC website are Stanford’s *Emergency Manual* and Ariadne Labs’ *Operating Room Crisis Checklists* among multiple others which are the culmination of many years of study, research, simulation testing, revision and international collaboration. They include diagnosis and treatment steps for the most common crises of perioperative medicine and are proving to be useful tools in the hands of healthcare professionals to augment the quality of both education and response to real-life crises.

## Review

Why perioperative emergency manual use is needed

According to Harvard studies, failure to adhere to lifesaving processes of care was shown to be four-six times higher without use of an EM compared to when an EM was utilized [14]. One theory against the use of EMs is potential hindrance of treatment during time-sensitive events where immediate action is needed. However, aviation studies have shown that when an EM is combined with recurrent training, it can be used effectively during such events [12,15-17]. Furthermore, a simulation study from Virginia Mason Medical Center showed knowledge retention of required actions increases with EM usage (P=0.031) as does quality decision-making and proper use of protocol with almost two times the amount of performed tasks completed, with even greater adherence when a dedicated EM “reader” is integrated as part of the care team [2,15,18-20]. Another question is of EM efficacy during critical events wherein the diagnosis does not exactly align with those published in an EM. For the answer to this question we must refer to the above discussed definition of an EM as a complement resource to clinician knowledge and expertise, not an all-encompassing solution [2].

We need more research to further demonstrate EM use as a positive intellectual skill for every level of anesthesia providers in real life situations to further demonstrate improved patient care. Suggested ideas include integrating the practice into residency training and continuing education to increase awareness and usability not only to include intraoperative emergency care, but also pre-crisis education, peri-procedural care outside the OR, and post event debriefing [12,15].

Implementation worldwide

There are many examples of international awareness on this topic, again with overwhelming inference of the benefit that EM provide in patient care and safety. Prominent examples include but are not limited to The European Society of Anesthesiologists that promotes an EM throughout Europe, Sociedad Columbiana de Anestesiologia y Reanimacion translation and implementation in Columbia of the EM from Ariadne labs and Harvard, The Chinese Society of Anesthesiology and The Chinese Association of Anesthesiologists promote multiple EM translations in China, Guidelines for the Management of Critical Hemorrhage in Japan, The Australian Patient Safety Foundation created the Critical Medical Management manual. Implementation in China seems to be the largest and most in-depth overseas movement and will be discussed here as recognition of potential benefit in terms of exemplary implementation and a potential source of research information over time.

Translations of Stanford EM, Ariadne Lab Operating Room Crisis Checklists, and SPA Crisis Checklists were created in Chinese for a national implementation project in China. Presentations and didactic sessions were given in hospitals and national conferences and were aired online in the largest national anesthesia forum in China [21]. Copies of the EM were placed at every anesthesia station in the participating hospitals. The translated EM were published in December 2015 as free resources and in the first 6 months there were 125,000 manuals downloaded directly from the website. Nationwide simulation-based training was implemented in several large prominent Chinese hospitals starting with the training of those people holding leadership positions. This training yielded a 97% commitment from participants to organize EM simulation training in their respective hospitals with rapid fruition in 40% of those committed institutions after just two months [22]. One year after multi-institutional implementation a study was performed which shows strong correlation of EM usage to simulation training with roughly 69% of surveyed participant respondents having participated in multidisciplinary simulation training with 70%. These participants reported EM use during at least one critical event in the previous six months with the average number of events being two, proving a largely successful implementation plan and execution as a whole [23]. Additional planning and successful EM implementation through simulation training was completed in 2017 in the form of “Simulation Wars”, a competition of sorts including teams, a point system with judges, and awards. Analysis performed one year after the competition showed that EM usage during actual critical events increased significantly with 85% of survey respondents reporting EM usage in at least one odds ratio (OR) critical event [24].

Methods to increase emergency manual utilization

Many articles mention the need for EM on the basis of human error being a natural occurrence, and reliance on memorization alone futile. One article in particular from the Stanford team gives an in depth review on the psychology of memory recall in critical events as a basis to promote EM usage in order to apply best known practices for critical events [12]. They give a recommendable 4-category strategy for EM implementation as well: Create, Familiarize, Use, and Integrate.

Create: There is no need to invent the wheel. Many useful EM have been created using a range of formats including interactive versions and recommended use with large screens and tablets [4,25]. One prime example of an interactive application was created by the SPA and made accessible on wireless devices [26]. Likely, the largest accumulation of EM free for download is found on the EMIC website [27].

Familiarize: Familiarization does not only constitute EM in accessible places in ORs but also incorporation through training mechanisms, cultural acceptance, and planned clinical use [28-29]. Stanford suggests introducing EM in the first year of residency as well as introducing all OR personnel to the EM of choice [30]. Studies from programs at Vanderbilt, Case Western, and University of Chicago imply necessity not only to include event training, but also specific training on how to best use the EM [31-32]. A review article from Queensland, Australia agrees that this is the way to obtain the maximum benefit from each aid as such training will assist in avoiding a “distraction” factor from patient evaluation and treatment [33].

Use: Possibly the best proof in perioperative literature so far is that of implementation in China outlined above. Evidence showed that there are many methods to increase EM utilization through simulation including active training involvement in simulations, competition, demonstrations, workshops and short term training courses [34]. EM accessibility, location, and format are also seen as important factors. As these variables may differ between institutions, individualized institution-specific training involving simulation which includes these variables will be likely to ameliorate such differences.

Integrate: A workshop that was sponsored by the Anesthesia Patient Safety Foundation (APSF) recommended to increase education and EM advocacy with the suggestion to include verification of EM presence in the pre-surgical time out and a reader be designated at that time [5]. Nationwide VHA integration reinforced the fact that EM placement in an OR is not sufficient for complete integration into the local culture. Merely 7% of respondents had used the aid in an emergency 6 months after EM placement despite 87% being aware of the presence of the EM [35]. This example contrasts starkly to the strategy seen in China with 70% of all respondents reporting usage in critical events after 6 months with high correlation to multidisciplinary simulation training. Leadership engagement and local champions are also key to success for EM integration through a long and arduous process that may take years and much effort such as it took in other professions where EM are commonplace now [2,36].

Emergency manuals improve patient safety

Communication breakdown and information loss as well as increased workload and competing tasks pose the greatest threats to patient safety in the OR [37]. EM integration can harbor a culture of teamwork where tasks are delegated and workload shared with open communication and memory stimulation through the use of EM, therefore benefiting patients and promoting safety as shown by a multi-institutional study wherein medical team training lowered annual mortality by 18% [38]. Many studies using simulation scenarios have shown that integrated use of EM catch errors and oversights that could potentially cause harm to patients and improve management of critical events [4,8]. One simulation study on ST-elevation myocardial infarction management during caesarean section showed improved task performance of participants by over 24% [15]. Similarly, simulation of an unstable intensive care unit patient demonstrated 23% improvement in critical event management based on tasks completed [39]. Additionally, potentially 100% of harmful oversights are overcome when teamwork is expanded even further with the addition of a “reader” as seen in a 2012 publication of obstetric cardiac arrest and mental health (MH) [20].

Prime examples of EM directly affecting patient safety in the perioperative arena also include Surgical Safety Checklists (SSC) that have been shown through multiple studies to significantly reduce rates of death and complications by more than 50% [38,40-42]. One study representing over 12 thousand patients from 76 countries demonstrated a lowered 30 day perioperative mortality [43]. A meta-analysis representing over 35,000 patients indicated that use of checklists led to a significant reduction in complications including wound infection and blood loss [44]. Additionally, a more recent retrospective review on over 21,000 surgical procedures not only demonstrated decreased mortality rates with use of the World Health Organization’s SSC, but also a decrease in length of admission by nearly 10% [45]. However, unless EM are used with active positive engagement coupled with strong implementation, it is unlikely to be successful [36].

According to a systematic review of crisis resource management skills learned in simulation training can transfer to clinical settings and lead to improved patient outcomes [46]. Examples of improved patient outcomes can perhaps be exemplified by the following four case reports: successful real-time use of the Harvard crisis checklist for air embolism in 2012 [47], a similar case using the Stanford EM at West Virginia University showed successful real-time use in an infant undergoing cranioplasty that required treatment for MH in 2014 [48]. Members of the Stanford team recently produced a case study in 2018 showing improved delivery of evidence-based patient care through effective teamwork using the EM as a complement to clinical knowledge and judgement in treatment of intraoperative cardiac arrest [49]. The first case report stemming from successful EM implementation in China in 2018 showed efficient use of an EM in the treatment of bronchospasm [50].

## Conclusions

Published works have thus far included a very high percentage of simulated scenarios and surveys due to the difficulty in measuring behaviors that are empowering for patient safety, such as improved communication, team-work, or identification of problems before they arise. The greatest resource on the topic of EM research in the future may be continued real-life case reports from an increasing amount of successful EM-implementing institutions detailing simulation training, usage, and positive outcomes in the form of increased patient safety. In conclusion, while effective implementation is challenging, there are successful examples on both large and small scales that, if utilized with a high level of compliance encouraged by local patient-safety champions, there is potential to save lives and quality of life which is the ultimate goal.

## References

[1] Hepner DL, Arriaga AF, Cooper JB (2017). Operating room crisis checklists and emergency manuals. Anesthesiology.

[2] Goldhaber-Fiebert SN, Macrae C (2018). Emergency manuals: how quality improvement and implementation science can enable better perioperative management during crises. Anesthesiol Clin.

[3] Gaba DM (2013). Perioperative cognitive aids in anesthesia: what, who, how, and why bother. Anesth Analg.

[4] Krombach JW, Marks JD, Dubowitz G, Radke OC (2015). Development and implementation of checklists for routine anesthesia care: a proposal for improving patient safety. Anesth Analg.

[5] Morell RC, Cooper JB (2018). APSF sponsors workshop on implementing emergency manuals. Anesthesia Patient Safety Foundation.

[6] Bittner D (2019). The power of cas for anesthesiologists in managing perioperative crises. MD Conf Express.

[7] Clay-Williams R, Colligan L (2015). Back to basics: checklists in aviation and healthcare. BMJ Qual Saf.

[8] Hagerman NS, Varughese AM, Kurth CD (2014). Quality and safety in pediatric anesthesia: how can guidelines, checklists, and initiatives improve the outcome?. Curr Opin Anaesthesiol.

[9] Moitra VK, Gabrielli A, Maccioli GA, O’Connor MF (2012). Anesthesia advanced circulatory life support. Can J Anesth Can d’anesthésie.

[10] Helmreich RL, Merritt AC, Wilhelm JA (1999). The evolution of crew resource management training in commercial aviation. Int J Aviat Psychol.

[11] Institute of Medicine (2000). To Err is Human: Building a Safer Health System. Washington, D.C.: National Academies Press.

[12] Goldhaber-Fiebert SN, Howard SK (2013). Implementing emergency manuals: can cognitive aids help translate best practices for patient care during acute events. Anesth Analg.

[13] Clebone A, Burian B, Watkins S, Gálvez J, Lockman J, Heitmiller E (2017). The development and implementation of cognitive aids for critical events in pediatric anesthesia: the society for pediatric anesthesia critical events checklists. Anesth Analg.

[14] Fowler AJ, Agha RA (2013). In response: simulation-based trial of surgical-crisis checklists. Ann Med Surg.

[15] St.Pierre M, Luetcke B, Strembski D, Schmitt C, Breuer G (2017). The effect of an electronic cognitive aid on the management of st-elevation myocardial infarction during caesarean section: a prospective randomised simulation study. BMC Anesthesiol.

[16] Burian BK (2006). Design guidance for emergency and abnormal checklists in aviation. Proc Hum Factors Ergon Soc Annu Meet.

[17] Burian BK (2019). Emergency and abnormal checklist design factors influencing flight crew response: a case study. Sage Journals. Proceedings of the Human Factors and Ergonomics Society Annual Meeting.

[18] Neal JM, Hsiung RL, Mulroy MF, Halpern BB, Dragnich AD, Slee AE (2012). ASRA checklist improves trainee performance during a simulated episode of local anesthetic systemic toxicity. Reg Anesth Pain Med.

[19] Marshall SD, Mehra R (2014). The effects of a displayed cognitive aid on non-technical skills in a simulated can’t intubate, can’t oxygenate crisis. Anaesthesia.

[20] Burden AR, Carr ZJ, Staman GW, Littman JJ, Torjman MC (2012). Does every code need a “reader?” improvement of rare event management with a cognitive aid “reader” during a simulated emergency: a pilot study. Simul Healthc.

[21] Huang JM (2018). Implementation of emergency manuals in china. Anesthesia Patient Safety Foundation. APSF.

[22] Huang JM (2019). Successful implementation of a two-hour emergency manual (EM) simulation instructor training course for anesthesia professionals in China. J APSF.

[23] Huang J, Wu J, Dai C (2018). Use of emergency manuals during actual critical events in china: a multi-institutional study. Simul Healthc: J Soc Simul Healthc.

[24] Huang J, Parus A, Wu J, Zhang C (2018). Simulation competition enhances emergency manual uses during actual critical events. Cureus.

[25] Wu L, Cirimele J, Card S, Klemmer S, Chu L, Harrison K (2011). Maintaining shared mental models in anesthesia crisis care with nurse tablet input and large-screen displays. Poster at UIST: ACM Symposium on User Interface Software and Technology..

[26] (2019). Quality & Safety Committee | Society for Pediatric Anesthesia. https://www.pedsanesthesia.org/critical-events-checklist/..

[27] (2018). EMIC Free Tools. https://www.emergencymanuals.org/tools-resources/free-tools/.

[28] Goldhaber-Fiebert SN, Lei V, Nandagopal K, Bereknyei S (2015). Emergency manual implementation: can brief simulation-based or staff trainings increase familiarity and planned clinical use. Jt Comm J Qual Patient Saf.

[29] Goldhaber-Fiebert SN, Howard SK (2013). Implementing emergency manuals: can cognitive aids help translate best practices for patient care during acute events?. Anesth Analg.

[30] Goldhaber-Fiebert SN, Pollock J, Howard SK, Merrell SB (2016). Emergency manual uses during actual critical events and changes in safety culture from the perspective of anesthesia residents: a pilot study. Anesth Analg.

[31] Watkins SC, Anders S, Clebone A (2016). Mode of information delivery does not effect anesthesia trainee performance during simulated perioperative pediatric critical events: a trial of paper versus electronic cognitive aids. Simul Healthc.

[32] Watkins SC, Anders S, Clebone A (2016). Paper or plastic? Simulation based evaluation of two versions of a cognitive aid for managing pediatric peri-operative critical events by anesthesia trainees: evaluation of the society for pediatric anesthesia emergency checklist. J Clin Monit Comput.

[33] Marshall S (2013). The use of cognitive aids during emergencies in anesthesia: a review of the literature. Anesth Analg.

[34] Huang JM (2019). New ways explored to promote emergency manual simulation training in China. NEWLETTER - Off J APSF.

[35] Neily J, DeRosier JM, Mills PD, Bishop MJ, Weeks WB, Bagian JP (2019). Awareness and use of a cognitive aid for anesthesiology. Jt Comm J Qual Patient Saf.

[36] Banguti PR, Mvukiyehe JP, Durieux ME (2018). The world health organization surgical safety checklist: happy 10th birthday. Anesth Analg.

[37] Christian CK, Gustafson ML, Roth EM (2006). A prospective study of patient safety in the operating room. Surgery.

[38] Neily J, Mills PD, Young-Xu Y (2010). Association between implementation of a medical team training program and surgical mortality. JAMA.

[39] Kazior MR, Wang J, Stiegler MP, Nguyen D, Rebel A, Isaak RS (2019). Emergency manuals improved novice physician performance during simulated ICU emergencies. J Educ Perioper Med JEPM.

[40] Haynes AB, Weiser TG, Berry WR (2009). A surgical safety checklist to reduce morbidity and mortality in a global population. N Engl J Med.

[41] de Vries EN, Prins HA, Crolla RMPH (2010). Effect of a comprehensive surgical safety system on patient outcomes. N Engl J Med.

[42] Bliss LA, Ross-Richardson CB, Sanzari LJ, Shapiro DS, Lukianoff AE, Bernstein BA, Ellner SJ (2012). Thirty-day outcomes support implementation of a surgical safety checklist. J Am Coll Surg.

[43] GlobalSurg Collaborative (2019). Pooled analysis of WHO surgical safety checklist use and mortality after emergency laparotomy. Br J Surg.

[44] Gillespie BM, Chaboyer W, Thalib L, John M, Fairweather N, Slater K (2014). Effect of using a safety checklist on patient complications after surgery: a systematic review and meta-analysis. Anesthesiology.

[45] de Jager E, Gunnarsson R, Ho Y-H (2019). Implementation of the world health organization surgical safety checklist correlates with reduced surgical mortality and length of hospital admission in a high-income country. World J Surg.

[46] Boet S, Bould MD, Fung L (2014). Transfer of learning and patient outcome in simulated crisis resource management: a systematic review. Can J Anesth.

[47] Ramirez M, Grantham C (2012). Crisis checklists for the operating room, not with a simulator. J Am Coll Surg.

[48] Ranganathan P, Phillips JH, Attaallah AF, Vallejo MC (2014). The use of cognitive aid checklist leading to successful treatment of malignant hyperthermia in an infant undergoing cranioplasty. Anesth Analg.

[49] Bereknyei Merrell S, Gaba DM, Agarwala AV (2018). Use of an emergency manual during an intraoperative cardiac arrest by an interprofessional team: a positive-exemplar case study of a new patient safety tool. Jt Comm J Qual patient Saf.

[50] Huang J, Jiang F, Zhang JF, Li MQ (2019). The use of emergency manuals leading to successful treatment of severe bronchospasm. J Med Pr Manag.

